# Lethal Congenital Contracture Syndrome 11: A Case Report and Literature Review

**DOI:** 10.3390/jcm11133570

**Published:** 2022-06-21

**Authors:** Miriam Potrony, Antoni Borrell, Narcís Masoller, Alfons Nadal, Leonardo Rodriguez-Carunchio, Karmele Saez de Gordoa Elizalde, Juan Francisco Quesada-Espinosa, Jose Luis Villanueva-Cañas, Montse Pauta, Meritxell Jodar, Irene Madrigal, Celia Badenas, Maria Isabel Alvarez-Mora, Laia Rodriguez-Revenga

**Affiliations:** 1 Biochemistry and Molecular Genetics Department, Hospital Clinic of Barcelona, 08036 Barcelona, Spain; potrony@clinic.cat (M.P.); mjodar@clinic.cat (M.J.); imadbajo@clinic.cat (I.M.); cbadenas@clinic.cat (C.B.); mialvarez@clinic.cat (M.I.A.-M.); 2 CIBER of Rare Diseases (CIBERER), Instituto de Salud Carlos III, 28029 Madrid, Spain; aborrell@clinic.cat (A.B.); masoller@clinic.cat (N.M.); 3 Institut d’Investigacions Biomèdiques August Pi i Sunyer (IDIBAPS), 08036 Barcelona, Spain; anadal@clinic.cat (A.N.); mpauta@clinic.cat (M.P.); 4 BCNatal, Barcelona Center for Maternal-Fetal and Neonatal Medicine (Hospital Clínic and Hospital Sant Joan de Deu), Institut Clínic de Ginecologia, Obstetricia i Neonatologia Fetal i+D Fetal Medicine Research Center, Universitat de Barcelona, 08007 Barcelona, Spain; 5 Pathology Department, Biomedical Diagnostic Center Hospital Clínic de Barcelona, 08036 Barcelona, Spain; lerodrig@clinic.cat (L.R.-C.); saezdegord@clinic.cat (K.S.d.G.E.); 6 Department of Basic Clinical Practice, Medical School, Universitat de Barcelona, 08007 Barcelona, Spain; 7 Medicine Department, University of Vic-Central University of Catalonia (UVic-UCC), 08500 Barcelona, Spain; 8 Genetics Department, 12 de Octubre University Hospital, 28041 Madrid, Spain; juanf.quesada@ingene.es; 9 UDISGEN (Unidad de Dismorfología y Genética), 12 de Octubre University Hospital, 28041 Madrid, Spain; 10 Molecular Biology CORE (CDB), Hospital Clínic de Barcelona, 08036 Barcelona, Spain; jlvillanueva@clinic.cat

**Keywords:** *GLDN*, arthrogryposis multiplex congenita, fetal akinesia deformation sequence

## Abstract

Lethal congenital contracture syndrome 11 (LCCS11) is caused by homozygous or compound heterozygous variants in the *GLDN* gene on chromosome 15q21. *GLDN* encodes gliomedin, a protein required for the formation of the nodes of Ranvier and development of the human peripheral nervous system. We report a fetus with ultrasound alterations detected at 28 weeks of gestation. The fetus exhibited hydrops, short long bones, fixed limb joints, absent fetal movements, and polyhydramnios. The pregnancy was terminated and postmortem studies confirmed the prenatal findings: distal arthrogryposis, fetal growth restriction, pulmonary hypoplasia, and retrognathia. The fetus had a normal chromosomal microarray analysis. Exome sequencing revealed two novel compound heterozygous variants in the *GLDN* associated with LCCS11. This manuscript reports this case and performs a literature review of all published LCCS11 cases.

## 1. Introduction

Arthrogryposis is characterized by congenital joint contractures in two or more body areas resulting from reduced or absent fetal movements [1]. Once the contracture is formed, a variety of secondary deformations occur, including craniofacial changes, pulmonary hypoplasia, polyhydramnios, decreased gut mobility and shortened gut, short umbilical cord, skin changes, and multiple joints with limitation of movement. Arthrogryposis is a complex trait that exhibits phenotypic and genotypic heterogeneity with an overall incidence of 1 in 3000 to 5000 [2]. Rather than a diagnosis, arthrogryposis is a descriptive term since it encompasses more than 400 medical conditions [3]. Alternative nomenclature in the literature includes multiple congenital contractures (MCC), arthrogryposis multiplex congenita (AMC), and fetal akinesia deformation sequence (FADS) or Pena–Shokeir syndrome type I (reviewed in [4]). Prenatal ultrasound imaging is crucial in its early diagnosis by identifying fetal movement limitations and the presence of club foot or joint contractures [5]. On prenatal suspicion of arthrogryposis, genetic diagnosis is important not only for identifying the causative genetic variant(s), but also for genetic counseling in regard to the prognosis, recurrence risk, and the options of prenatal testing or reproductive choice for future pregnancies.

The use of next-generation sequencing (NGS) methods in the diagnostic workup of arthrogryposis has proved to be an efficient technology in achieving the underlying genetic causes in many cases, i.e., [6,7,8]. The diagnosis rates of arthrogryposis improve up to 60% when whole-exome sequencing (WES) is used [8]. In fact, this strategy has also allowed the identification of new arthrogryposis-associated genes such as *GLDN* [9].

The *GLDN* gene encodes the gliomedin protein, a secreted cell adhesion molecule involved in peripheral nervous system development. Biallelic variants in the *GLDN* gene have recently been associated with lethal congenital contracture syndrome 11 (LCCS11, OMIM # 617194), a clinically severe form of AMC [9,10]. Here, we report a prenatal diagnosis of LCCS11 detected by WES in a fetus with AMC, hydrops, and retrognathia, and a literature review of all cases reported to date. Although *GLDN* has been described as a new AMC-associated gene, we conclude that it should be better associated with FADS or Pena–Shokeir syndrome type I.

## 2. Case Report

**Figure 1:**
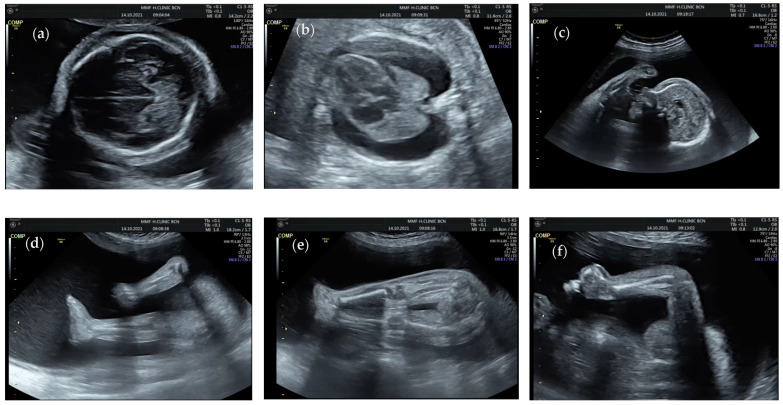
Transabdominal ultrasound images of the present case. Transabdominal ultrasound images of the present case showing (**a**) scalp edema, (**b**) subcutaneous edema and hydrothorax, (**c**) forehead edema, (**d**,**e**) lower extremity hyperextension, (**f**) upper extremity and hand contracture.

A 35-year-old primigravid woman was referred at the 28th week of gestation for hydrops fetalis and arthrogryposis. Sonography examination revealed hydrothorax, subcutaneous generalized edema, short long bones, fixed limb joints, absent fetal movements, fetal growth restriction (estimated fetal weight in the 4th percentile and absent end-diastolic flow in both umbilical arteries), and polyhydramnios (amniotic fluid index 28 cm) (Figure 1). The couple was nonconsanguineous, healthy, and both showed unremarkable family history with no congenital malformations. The mother denied any exposure to alcohol, teratogenic agents, irradiation, or infectious diseases during this pregnancy. Serologic testing for TORCH (Toxoplasmosis, Rubella, Cytomegalovirus, Herpes simplex virus) infection diseases was negative. In consideration of the abnormal ultrasound findings, amniocentesis was performed and chromosomal microarray analysis (CMA) was performed using qChipPrenatal microarray (qGenomics, Spain) on uncultured amniocytes. The qChipPrenatal microarray is a genome-wide oligonucleotide array (based on an Agilent 8 × 60 K format) with a practical resolution of approximately 350–500 Kb throughout the entire genome and 30–100 Kb in regions associated with constitutional pathology (qChipCM, 8 × 60 K, qGenomics). The results revealed a normal female profile, arr(X, 1 − 22) × 2. Written informed consent was obtained from the pregnant woman.

**Figure 2:**
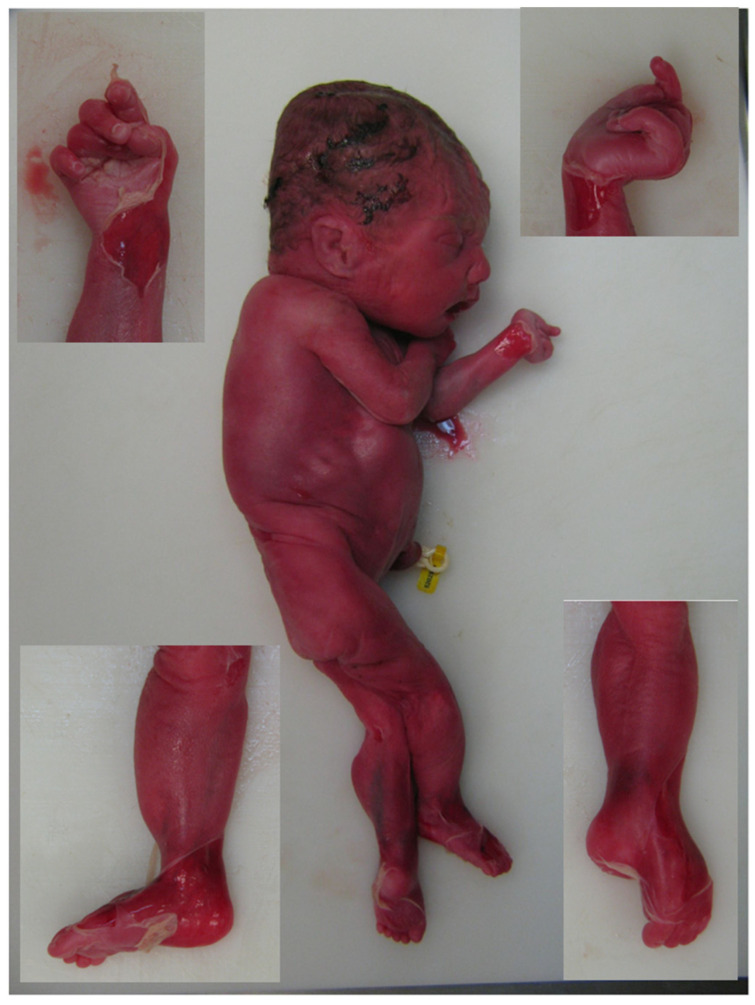
Lateral view of the fetus. Lateral view of the fetus shows skin slippage due to maceration. Both hands show medially overlapping fingers (**upper insets**) and left pes equinus (**lower insets**).

The woman elected to terminate the pregnancy at 29 weeks of gestation. Postmortem examination was performed and findings were consistent with the prenatally observed sonographic anomalies. The autopsy revealed a slightly macerated female fetus with hydrops with subcutaneous edema and pleural effusions, distal arthrogryposis of the hands, left pes equinus, flexed elbows with preserved mobility of all major joints, fetal growth restriction, pulmonary hypoplasia with a lung to body weight ratio of 0.0058 (normal > 0.012), and retrognathia (Figure 2). Histological examination of the brain was unremarkable.

WES analysis was further performed. Massively parallel sequencing was performed using DNA Prep with Enrichment (Exome capture, Illumina, San Diego, CA, USA) on a NextSeq 500 sequencer (Illumina, San Diego, CA, USA), with a targeted mean coverage of 100× and a minimum of 90% of bases sequenced to at least 20×. Bioinformatic analysis consisted of alignment to the reference human genome (hg38) using BWA MEM (v0.7.17) and Bowtie2 (v2.4.1) short-read aligners, genotyping using Haplotype Caller from Genome Analysis Toolkit (v.4.2) and VarDict (v1.7.0) variant callers, and annotation using Ensembl Variant Effect Predictor (v104). Copy Number Variants (CNVs) analysis was performed using ExomeDepth R package (v1.15) for CNVs identification and AnnotSV (v2.3) for CNVs annotation. Variants that did not meet the established quality criteria were filtered out: strand bias variants or those in repetitive or high CGs content regions with low mapping quality reads. In addition, variants with frequency greater than 3% in gnomAD population database (v3.1.1) were also filtered together with those classified as benign or likely benign by multiple subscribers in the ClinVar database (March 2020 release). Variant interpretation and classification were performed according to the ACMG recommendations [11].

Results evidenced a compound heterozygous for two variants in the *GLDN* (NM_181789) gene. The maternally inherited *GLDN* variant (c.1494G>T, p.Leu498Phe) is a missense variant predicted to be damaging by the majority of in silico functional prediction programs (PolyPhen, SIFT, CADD, Mutation Taster). The leucine residue at this position has a high conservation score (phyloP and phastCons 100 vertebrates) and it is located within the conserved extracellular olfactomedin domain of gliomedin. The variant is absent in population databases (gnomAD, 1000G) and the same amino acid change has been previously reported in one LCCS11 case [12].

The paternally inherited variant is also a missense variant, c.62C>A, p.Ala21Glu, that has been detected in very low frequency in the general population (gnomAD: 4 heterozygous individuals, allele frequency 0.000058, dbSNP: rs778094534), but has not been previously detected in LCCS11-affected individuals. The affected alanine residue is partially conserved (phyloP and phastCons 100 vertebrates) and it is located within a trasmembrane domain. Although this variant did not have sufficient evidence to be classified as pathogenic in the absence of additional functional data, the phenotype of our patient is remarkably similar to that previously reported.

**Figure 3:**
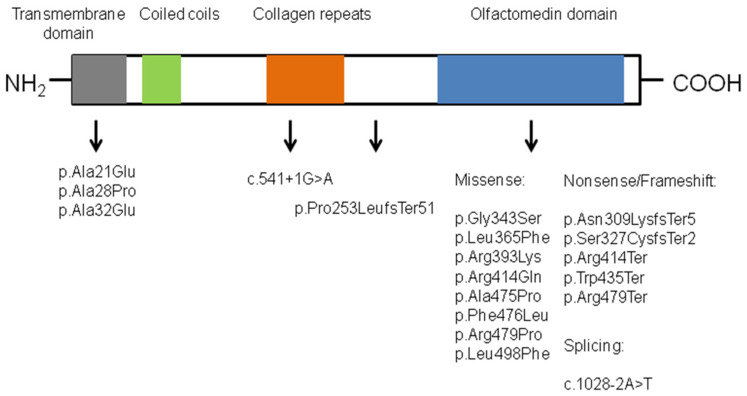
Location of the pathogenic/likely pathogenic variants identified in *GLDN* in AMC-affected families relative to the predicted protein domains.

The publications available in the literature were reviewed, and 28 cases, belonging to 19 different families, with compound heterozygous or homozygous variants in *GLDN,* were collected in this report (Figure 3). Table 1 summarizes the sonographic, postmortem, and molecular findings.

## 3. Discussion

Biallelic *GLDN* variants have been associated with a lethal form of AMC since most of the reported patients did not survive past neonatal ages (LCCS11) [9]. However, among the 28 herein reviewed cases, there are 6 long-term survivors (from 5 families) that, although the majority required intensive clinical support, survived beyond the neonatal period [7,10,14]. On the basis of these cases, it has been suggested that pulmonary insufficiency in patients with biallelic *GLDN* variants is not necessarily lethal [10,14]. Nevertheless, 57% (8/14) of the neonate cases died before 2 months. The remaining six cases survived beyond the neonatal period although they required intensive respiratory support.

A distinguishing clinical feature described in the majority of patients with pathogenic *GLDN* variants is pulmonary hypoplasia. To our knowledge, among the herein 28 reviewed cases, 16 reported respiratory findings, pulmonary hypoplasia being the most frequent (75%, 12/16), followed by pulmonary insufficiency or need of respiratory support. As pulmonary hypoplasia is a feature not common in AMC at large, some authors have recently suggested that AMC secondary to *GLDN* variants may be best fitted under the umbrella of FADS [14]. The FADS (ORHA:994) is characterized by multiple joint contractures, facial anomalies, and pulmonary hypoplasia. The common feature of this sequence is decreased fetal activity, which leads to a failure of normal deglutition, resulting in polyhydramnios. The lack of movement of the diaphragm and intercostal muscles leads to pulmonary hypoplasia. Finally, the lack of normal fetal movement also results in a short umbilical cord and multiple joint contractures.

Sonographic detection of AMC in a prenatal context is often missed or diagnosed during late gestation, when associated anomalies are more pronounced [18,19]. In the series herein reviewed, approximately 30–32 weeks of gestational age is the mean gestational age of prenatal diagnosis, with fetal akinesia, missing fetal movements, arthrogryposis, and polyhydramnios being the most frequently reported features. Among the 28 reviewed cases, 29% (8/28) elected to terminate pregnancy. Postmortem examination is only reported in half of them, confirming the prenatal diagnosis and expanding the associated phenotype spectrum with pulmonary hypoplasia, retrognathia, and clubfoot (Table 1).

Due to the relative rarity of this entity, few patients have been reported; this makes it difficult to establish a genotype–phenotype correlation. Among the 19 different pathogenic variants described in the *GLDN* gene (Table 1), the majority of them (68%, 13/19) correspond to missense, nonsense, or frameshift variants located within the highly conserved olfactomedin domain (aa 300–550) [20] (Figure 3). The olfactomedin domain mediates the interaction between gliomedin and NrCAM, as well as neurofascin-186 (NF186), two cell adhesion molecules expressed at the nodes of Ranvier, to induce clustering of sodium channels at heminodes of myelinating Schwann cells [20,21,22,23]. Thus, these variants might impact the formation of the NrCAM–NF186–gliomedin complex at nodes. To our knowledge, only three different missense variants (c.95C>A, c.82G>C and the c.62C>A detected in the present case) have been described outside this domain and within the transmembrane domain of gliomedin (aa 16–38) (Table 1, Figure 3) [21,22]. Although these variants might be initially classified as variants of uncertain significance (VUS), as the amino acid residues are not highly conserved, functional analyses have also revealed an abnormal localization of the resultant protein [9,14]. Western blotting experiments in transfected CHO cells with different *GLDN* variants showed similar amounts of GLDN protein [9]. Thus, it can be inferred that rather than a loss of function effect, pathogenic variants detected in the *GLDN* gene affect gliomedin’s transportation to the cell surface and its binding to NF186 [9,14].

## 4. Conclusions

The present reported case and the literature review confirms the association of biallelic *GLDN* variants with AMC and other phenotypic spectra such as pulmonary hypoplasia, reaffirming that it should be better classified as FADS. Prenatal diagnosis of this condition is challenging since it is often missed or diagnosed in the second or third trimester. Postnatal autopsy is recommended as it confirms the prenatal diagnosis and might identify further associated congenital anomalies. Furthermore, it provides a valuable source of DNA material. Finally, and due to the high degree of genetic heterogeneity, WES should be recommended when a FADS is suspected. Once the underlying etiology is known, genetics consultation and individualized recurrence risk assessment can be offered.

## Figures and Tables

**Table 1:** Clinical characteristics of cases with biallelic *GLDN* variants and arthrogryposis multiplex congenita (AMC).

ID	Sex	Prenatal Ultrasound Examination	Fetal Death	Postmortem Examination	Birth	Genetic Variant 1	Genetic Variant 2	Reference
Family 1 Case 1	male	32 wg: Akinesia Polyhydramnios	Exitus 33 wg	Extension of lower limbs Extension contractures of wrists Pulmonary hypoplasia	-	c.758delC p.(Pro253LeufsTer51)	c.1423G>C p.(Ala475Pro)	[9]
Family 1 Case 2	female	Akinesia Polyhydramnios	TOP 33 wg	Unremarkable histological examination of the spinal cord and skeletal muscle Reduced number of myelinated fibers	-	c.758delC p.(Pro253LeufsTer51)	c.1423G>C p.(Ala475Pro)	[9]
Family 2 Case 1	male	30 wg Polyhydramnios Intrauterine growth retardation AMC (flexion contractures of the elbows, extension of the knees, camptodactyly, and retrognathia)	-	NI	30 wg AMC (flexion contractures of the elbows, extension of the knees, camptodactyly, and retrognathia) Exitus: day 1	c.95C>A p.(Ala32Glu)	c.95C>A p.(Ala32Glu)	[9]
Family 3 Case 1	male	28 wg: Akinesia Polyhydramnios Bilateral flexion of fingers	-	Unremarkable pathological examination of the brain and spinal cord	AMC (involving the fingers, wrists, thumbs, and knees) Pulmonary hypoplasia Exitus: day 1	c.541 + 1G>A	c.1240C>T p.(Arg414Ter)	[9]
Family 3 Case 2	male	31 wg: Polyhydramnios Bilateral flexion of fingers Reduced mobility	TOP 31 wg	AMC with microretrognathia Pulmonary hypoplasia	-	c.541 + 1G>A	c.1240C>T p.(Arg414Ter)	[9]
Family 4 Case 1	female	27 wg: Reduced mobility Polyhydramnios 29 wg: Fetal Immobility	TOP 30 wg	Unremarkable pathological examination of the brain and spinal cord	Distal arthrogryposis of the hands Bilateral club foot Pulmonary hypoplasia	c.1435C>T p.(Arg479Ter)	c.1435C>T p.(Arg479Ter)	[9]
Family 5 Case 1	male	Reduced mobility Breech	-	AMC Pulmonary hypoplasia and pulmonary hemorrhage Bilateral hip dislocations Fistula from the left anterior descending artery to right ventricle Bilateral small kidneys with calcifications, an ectopic right ureter without signs of obstruction, and intraventricular hemorrhage Skeletal muscle fibers were small for age and central nuclei suggested centronuclear myopathy	38 wg Respiratory failure Exitus: day 2	c.927_930del p.(Asn309LysfsTer5)	c.1436G>C p.(Arg479Pro)	[10]
Family 5 Case 2	female	Polyhydramnios Intrauterine growth restriction Bilateral club feet	-	-	37 wg Respiratory insufficiency Contractures of hips, knees fixed in extension Bilateral club feet Flexion contracture of left long finger Bilateral hip dislocation Axial and appendicular hypotonia Alive at 22 months with tracheostomy and home ventilation	c.927_930del p.(Asn309LysfsTer5)	c.1436G>C p.(Arg479Pro)	[10]
Family 5 Case 3	male	Polyhydramnios Bilateral club feet Flexed wrists Extended knees Breech Intrauterine growth restriction	-	-	39 wg Respiratory insufficiency Contractures of hips, knees Bilateral club feet Hyperextension of thumbs to radii Axial and appendicular hypotonia Undescended testes Alive at 7 months with tracheostomy and home ventilation	c.927_930del p.(Asn309LysfsTer5)	c.1436G>C p.(Arg479Pro)	[10]
Family 6 Case 1	male	Polyhydramnios	-	-	33 wg Pulmonary hypoplasia Bilateral hip dislocation Contractures of knees and wrists Bilateral club feet Progressive scoliosis, diaphragm paralysis, borderline intellectual functioning (IQ 74) Alive at age 17 years old with intermittent use of non-invasive mask ventilation	c.1305G>A p.(Trp435Ter)	c.1305G>A p.(Trp435Ter)	[10]
Family 7 Case 1	female	30 wg. Akinesia Polyhidramnios Skin edema	TOP 31 wg	NI	-	c.1305G>A p.(Trp435Ter)	c.1305G>A p.(Trp435Ter)	[10]
Family 7 Case 2	male	-	-	-	41 wg Paresis of right vocal cord and right side of the soft palate Bilateral hip flexion contractures with dislocated hips Extension contractures of kneesCalcaneovalgus deformity of feet Axial and appendicular hypotonia Atrophy of lower limbs Right-sided cryptorchidism Intubated at birth for respiratory failure Tracheostomy at 6 weeks of age Alive at 28 months without ventilatory support	c.1305G>A p.(Trp435Ter)	c.1305G>A p.(Trp435Ter)	[10]
Family 8 Case 1	male	Akinesia Flexed arms and closed hand	TOP 27 wg	Pulmonary hypoplasia Extension contractures of hip sand knees Flexion contractures of fingers	-	Unknown	Unknown	[10]
Family 8 Case 2	female	26 wg: Polyhydramnios Arthrogryposis	-	-	36 wg: Pulmonary hypoplasia Extension contractures of hips and knees Flexion contractures of elbows, wrists, and fingers Bilateral vertical talus information Diffuse muscle atrophy/hypoplasia Exitus: 12 h	c.1178G>A p.(Arg393Lys)	c.1428C>A p.(Phe476Leu)	[10]
Family 9 Case 1	male	26 wg: Multiple joint contracture Polyhydramnios	-	-	-	c.1027G>A p.(Gly343Ser)	c.1240C>T p.(Arg414Ter)	[13]
Family 9 Case 2	female	26 wg: Multiple joint contracture Polyhydramnios	-	-	-	c.1027G>A p.(Gly343Ser)	c.1240C>T p.(Arg414Ter)	[13]
Family 10 Case 1	-	NI	NI	NI	NI	c.1494G>C p.(Leu498Phe)	c.1494G>C p.(Leu498Phe)	[12]
Family 11 Case 1	female	Early fetal demise of a twin <12 wg Polyhydramnios Preterm premature rupture of membranes Breech (20 wg)	-	-	30 wg: Bilateral extension knee contractures and camptodactyly Bilateral congenital hip dysplasia and right-sided hip dislocation Hypotonia Pulmonary hypoplasia Alive at 44 months	c.1093C>T p.(Leu365Phe)	c.1178G>A p.(Arg393Lys)	[14]
Family 12 Case 1	female	Fetal akinesia	NI	NI	Joint contractures: Hips, knees, ankles, elbows, fingers Microcephaly Delayed motor development Muscular hypertonia Hip joint luxation Alive at 1 year	c.1178G>A p.(Arg393Lys)	c.1428C>A p.(Phe476Leu)	[7]
Family 13 Case 1	male	Hydrops fetalis	-	-	Subtle joint contractures Down-slanted palpebral fissures Ventilator support Care redirected towards palliation	c.980_981del p.(Ser327CysfsTer2)	c.980_981del p.(Ser327CysfsTer2)	[15]
Family 14 Case 1	male	No findings	-	-	Exitus: < 1 month	c.95C>A p.(Ala32Glu)	c.95C>A p.(Ala32Glu)	[8] *
Family 15 Case 1	female	Abnormalities	TOP	NI		c.1435C>T p.(Arg479Ter)	c.1435C>T p.(Arg479Ter)	[8] *
Family 16 Case 1 + Case 2	Female (2 cases)	Abnormalities	-	NI	Exitus: 2 months	c.82G>C p.(Ala28Pro)	c.1241G>A p.(Arg414Gln)	[8] *
Family 17 Case 1	-	32 wg: Polyhydramnios Missing fetal movements Facial dismorphism Lung hypoplasia Flexed knees, extended anckles, flexed elbows, fisted hands	-	-	32 wg Exitus: 1 day	c.1423G>C p.(Ala475Pro)	c.1423G>C p.(Ala475Pro)	[16]
Family 17 Case 2	-	23 wg: Polyhydramnios Missing fetal movements Microcephaly Single umbilical artery Pericardial and pleural effusion Flexed knees, flexed elbows, fisted hands	TOP 27 wg	-	-	c.1423G>C p.(Ala475Pro)	c.1423G>C p.(Ala475Pro)	[16]
Family 18 Case 1	-	NI	-	-	Flexion contracture Hydrops fetalis Pulmonary hypoplasia Pleural effusion	c.1028-2A>T	c.1028-2A>T	[17]
PRESENT CASE	female	28 wg: Hydrops fetalis Arthrogryposis	TOP 29 wg	Distal arthrogryposis of the hands Left club foot Pulmonary hypoplasia Retrognathia	-	c.62C>A p.(Ala21Glu)	c.1494G > T p.(Leu498Phe)	PRESENT STUDY

wg: weeks of gestation; TOP: termination of pregnancy, NI: no information. * Cases already reported by Maluenda et al. [9] were excluded from this table. Families and cases have been renumbered in this table based on the order of appearance in each study.

## Data Availability

The analyzed data sets generated during the study are available from the corresponding author on reasonable request.
